# CT Findings in Negative Pressure Pulmonary Edema

**DOI:** 10.3390/diagnostics10100749

**Published:** 2020-09-25

**Authors:** Adrien Holzgreve, Matthias P. Fabritius, Philippe Conter

**Affiliations:** 1 Department of Nuclear Medicine, University Hospital, LMU Munich, 81377 Munich, Germany; 2 Department of Radiology, University Hospital, LMU Munich, 81377 Munich, Germany; Matthias.Fabritius@med.uni-muenchen.de; 3 Department of Anesthesiology, University Hospital, LMU Munich, 81377 Munich, Germany; Philippe.Conter@med.uni-muenchen.de

**Keywords:** negative pressure pulmonary edema (NPPE), computed tomography (CT), anesthesia complication, chest imaging, thoracic imaging, COVID-19 differential diagnosis

## Abstract

Negative pressure pulmonary edema (NPPE) is a rare, potentially life-threatening, and yet diagnostically challenging perioperative complication. Most cases of NPPE occur in the context of anesthetic procedures, mainly caused by upper airway obstruction, and are diagnosed during the recovery period. We present a case of fulminant NPPE in a patient during general anesthesia which illustrates the eye-catching CT findings that can occur in NPPE and eventually support diagnosis. With regard to the current pandemic, we include a discussion of the typical imaging patterns of COVID-19 as a radiological differential diagnosis of NPPE. A 42-year old male patient presented with sudden respiratory insufficiency during arthroscopic knee lavage and subsequently required highly invasive ventilation therapy and catecholamine administration. Postoperative CT imaging of the thorax exhibited extensive, centrally accentuated consolidations with surrounding ground-glass opacity in all lung lobes, suggestive of pulmonary edema. In view of the clinical course and the imaging findings, a negative pressure pulmonary edema (NPPE) was diagnosed.

**Figure 1:**
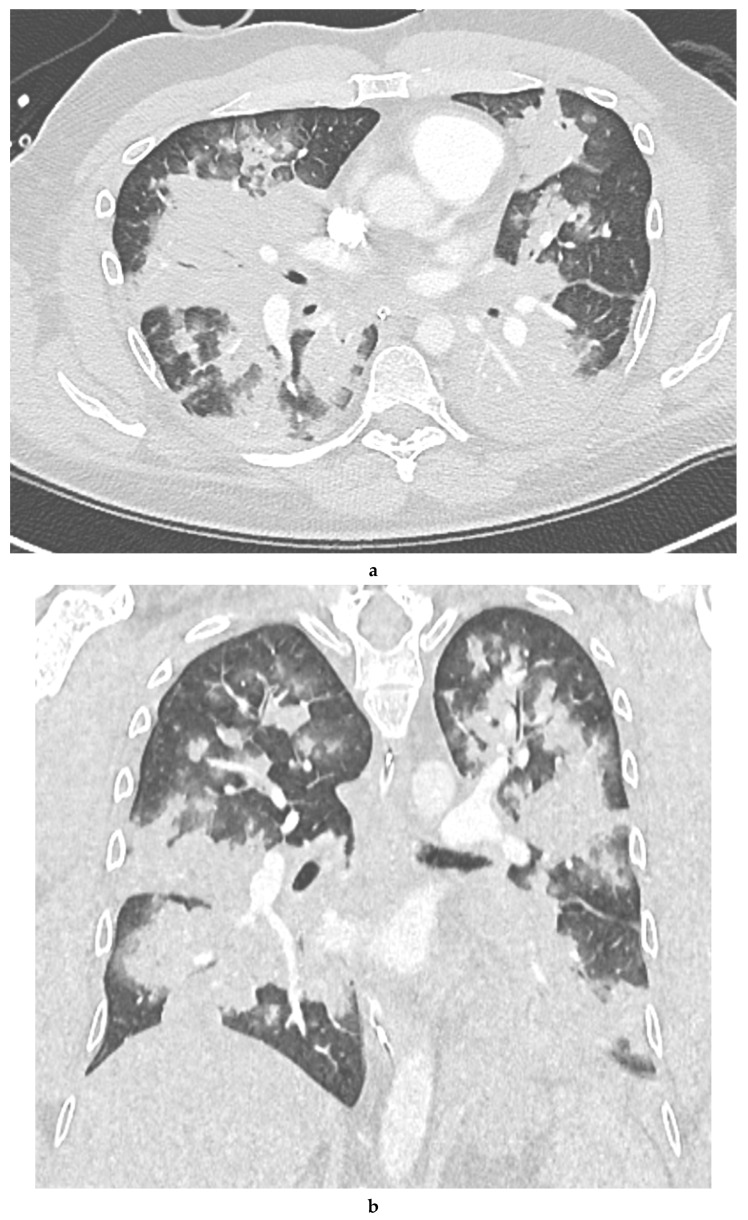
A 42-year-old male presented with petechiae and the deterioration of general condition. The initial laboratory workup was especially notable for hemoglobin 6.9 g/dL and thrombocytes 1 G/L. Laboratory testing was positive for Anti-GPIb/IX-antibodies, consistent with the diagnosis of primary immune thrombocytopenia. Dexamethasone for 3 days was started. Six days later, during hospitalization the patient developed an acute onset of knee pain and fever. Staphylococcus aureus was noted both in synovial fluid and blood culture and the decision for an arthroscopic knee lavage was made. In the operating room, after standard monitoring was established, general anesthesia was induced with an intravenous injection of sufentanil 30 mcg and propofol 230 mg. As ventilation via laryngeal mask was not sufficient, intubation was performed without problems after the intravenous injection of another propofol 50 mg and rocuronium 30 mg. Anesthesia was first maintained with propofol and sufentanil and then changed to sevoflurane and sufentanil. The administration of anesthesia was not controlled by depth-of-anesthesia monitoring. Ventilation therapy was initially performed with an oxygen fraction of 100%, positive end-expiratory pressure (PEEP) = 4 cm H_2_O, inspiratory pressure (Pinsp) = 21 cm H_2_O, and respiratory rate (RR) = 14/min. The oxygen fraction was subsequently reduced to 50%. Shortly after airway management and before incision, a respiratory decompensation with global insufficiency occurred and a highly invasive ventilation therapy was necessary, with an oxygen fraction of 100%, PEEP = 13 cm H_2_O, Pinsp = 40 cm H_2_O, and RR = 22/min. Arterial blood gas demonstrated a pH of 7.15, pO_2_ at 131.0 mmHg, pCO_2_ at 60.6 mmHg, bicarbonate at 18.1 mmol/L, base excess at −7.2 mmol/L, and lactate at 2.8 mmol/L. Because of insufficient lung compliance, a high driving pressure was necessary and lung protective ventilation could not be ensured. Anti-obstructive therapy with salbutamol and terbutaline showed no benefit. During endotracheal inspection, bloody-frothy secretion was noted. Furthermore, the initially normal blood pressure dropped after anesthesia induction (mean arterial pressure, MAP < 65 mmHg), and the patient required vasopressor support. Despite the administration of high-dose norepinephrine, hypotension persisted and vasopressin was added. There was no significant blood loss during arthroscopic surgery, yet the preoperative hemoglobin was 6.9 g/dL and one erythrocyte concentrate was administered. Additionally, there was no clinical reference for hypovolemia or drug overdose. After surgery (1 h), CT imaging of the thorax exhibited extensive, centrally accentuated consolidations with surrounding ground-glass opacity in all lung lobes, suggestive of pulmonary edema (see (**a**) for axial plane and (**b**) for coronal plane of the CT imaging of the thorax). Upon arrival in the ICU (5 h after anesthesia induction), ventilation therapy was performed with an oxygen fraction of 50%, PEEP = 10, Pinsp = 36, and RR = 24. Arterial blood gas demonstrated a pH of 7.29, pO_2_ at 69.0 mmHg, pCO_2_ at 48.7, bicarbonate at 21.4 mmol/L, base excess at −3.4 mmol/L, and lactate at 4.5 mmol/L. Norepinephrine administration was still required. Transesophageal echocardiography showed no pathological findings—i.e., no signs of hypovolemia or wall motion abnormalities. On the first postoperative day, catecholamine administration and ventilation therapy could be further deescalated to FiO_2_ = 35%, PEEP = 8, Pinsp = 17, and RR = 14. Arterial blood gas showed no pathological findings at this time. On the second postoperative day, ventilation therapy, sedation, and catecholamine administration were significantly reduced. Patient-triggered ventilation and the anesthetic recovery phase were uneventful, and the patient was extubated within 36 h. To maintain an oxygen saturation of >90%, the administration of oxygen was still necessary. After extubation, no catecholamine administration was required. The patient had no neurologic deficits. The following days in the ICU, noninvasive CPAP-therapy and intensive respiratory physiotherapy were performed to avoid pneumonic complications. The patient was transferred to general ward after 6 days in the ICU. In an ^18^F-FDG PET/CT examination performed 10 days later, the previously extensive bipulmonary findings had almost completely receded. The patient was discharged home in improved general condition 24 days after hospital admission and 18 days after the initial event. In view of the clinical course and the imaging findings, a negative pressure pulmonary edema (NPPE) was diagnosed. As NPPE is a complication of upper airway obstruction combined with forced inspiratory effort, difficult airway management was suspected to be the cause of the phenomenon in the current case. Differential diagnosis includes an acute exacerbation of pre-existing bronchial asthma, pulmonary embolism, and septic shock secondary to the septic gonarthritis. Differential diagnosis regarding the CT images also includes pneumonic infiltrates or massive aspiration. Pre-existing bronchial asthma was stable without any medication before hospital admission, and there was no clinical improvement after the administration of guideline therapy during respiratory insufficiency. Pulmonary embolism was ruled out via CT imaging. Pneumonic infiltrates were unlikely because of a chest x-ray with no pathological findings the day before surgery. Due to the current circumstances, the rapid aggravation of a previous clinical occult COVID-19 pneumonia has also been considered as a possible cause [1]. However, the imaging findings were not characteristic and COVID-19 was ruled out as standard on hospital admission and before surgery via PCR. During in-hospital stay, another three SARS-CoV-2-PCR tests were performed. They were all negative. In the ICU, septic shock was the most relevant differential diagnosis, thus it was treated by calculated sepsis therapy. NPPE is a rare and potentially life-threatening but well-described perioperative complication. NPPE develops after forced inspiratory efforts against simultaneously closed airways [2]. Several cases of this rare condition have been reported, however generally focusing on the underlying comorbidities [3,4,5,6,7,8] and not on the imaging findings. As many cases of NPPE are estimated to be undiagnosed due to a lack of familiarity with the syndrome and may therefore end fatally [9], we want to emphasize the contribution of imaging findings to this differential diagnosis. The current case illustrates the eye-catching imaging findings that can occur in NPPE, resulting in a fulminant non-cardiogenic bipulmonary edema. The ground-glass opacities and consolidations are also compatible with COVID-19 [10]; however, the predominantly centrally located pattern is not typical for COVID-19 [11], and the further clinical information including repeatedly negative SARS-CoV-2 PCR testing as well as the acute and sudden respiratory insufficiency after airway management, with albeit rapid clinical recovery, conflict with potential COVID-19 in the current case. In conclusion, NPPE can result in fulminant bipulmonary edema on CT, as illustrated by the present case. Albeit not mandatory for the clinical diagnosis of NPPE, CT imaging is able to contribute to the patient management in suspected NPPE—e.g., in ruling out important differential diagnosis, such as pulmonary embolism. In clinical routine, the occurrence of a pronounced bipulmonary edema following respiratory deterioration during general anesthesia should always lead to the rare differential diagnosis of a negative pressure pulmonary edema.
